# A pragmatic double blind remote pilot feasibility randomised controlled trial of a self-management app for people with Sjögren disease

**DOI:** 10.3389/fdgth.2025.1549093

**Published:** 2025-06-03

**Authors:** Katie L. Hackett, Miglena Campbell, Eduwin Pakpahan, John Vines, Dennis Lendrem, Jemma McCready, Tim Rapley, Jason Ellis, Vincent Deary, Elaine McColl, Claire McCallum

**Affiliations:** ^1^ Department of Social Work, Education and Community Wellbeing, Northumbria University, Newcastle upon Tyne, United Kingdom; ^2^ Institute for Collective Place Leadership, Teesside University, Middlesbrough, United Kingdom; ^3^ Department of Mathematics Physics and Electrical Engineering, Northumbria University, Newcastle upon Tyne, United Kingdom; ^4^ School of Informatics, University of Edinburgh, Edinburgh, United Kingdom; ^5^ Newcastle University Translational & Clinical Research Institute, Newcastle upon Tyne, United Kingdom; ^6^ Department of Psychology, Northumbria University, Newcastle upon Tyne, United Kingdom; ^7^ Population Health Sciences Institute, Newcastle University, Newcastle upon Tyne, United Kingdom; ^8^ College of Medical, Veterinary & Life Sciences, University of Glasgow, Glasgow, United Kingdom

**Keywords:** Sjögren's syndrome, Sjögren disease, smartphone app, feasibility, selfmanagement, tools

## Abstract

**Objectives:**

To pilot and assess the feasibility of a fully remote effectiveness evaluation of a novel smartphone self-management app for people living with Sjögren disease (SjD), including evaluating trial procedures and app engagement.

**Methods:**

We conducted a double-blind, randomised, fully-remote pilot feasibility of a self-management smartphone app (Sjogo) containing interactive components with an information-only control app. After completing onboarding procedures, participants were allocated to a trial arm following download from Apple App and Google Play stores. Participants completed symptoms and quality of life measures at baseline and (at two further timepoints (5–7 and 10–13 weeks) after download. Engagement with the app was measured with number and duration of logins.

**Results:**

996 participants downloaded Sjogo to their smartphone. 871 (87.45%) consented to take part in the study and 617 (61.95%) completed the onboarding procedures and baseline measures and were randomised to the full-version of the app (*n* = 318) or control-version (*n* = 299). In-app randomisation produced balanced groups. In week 1 engagement was higher in the intervention group *m* = 4.76 logins (S.D. 8.06) than the control group *m* = 3.47 (S.D. 2.75). At week 2 engagement dropped in both groups (intervention group *m* = 1.17, SD 4.56, control *m* = 0.40, SD 0.93). Outcome completion rates at subsequent timepoints were 36.63% (weeks 5–7) and 27.39% (weeks 10–13).

**Conclusion:**

It is feasible to collect data fully remotely, automate trial procedures, and recruit participants to a randomised controlled trial of a self-management smartphone app for people with SjD through app stores. However, app engagement and outcome completion rates could be improved.

## Introduction

Sjögren disease (SjD) is a common autoimmune disease with a prevalence rate of 65 per 100,000 and a female to male ratio incidence of 9–1 peaking around age 50 (1, 2). SjD is complex and while exocrinopathy causing ocular, oral and vaginal dryness is the main feature (3), SjD is also associated with fatigue, pain and sleep disturbances and impacts on daily activities and quality of life (4, 5). SjD has previously been defined as presenting independently [as primary Sjögren's Syndrome (SS)] or in association with another autoimmune disease, such as rheumatoid arthritis (RA) or systemic lupus erythematosus (SLE), and known as secondary SS (6).

Despite many SjD patients experiencing functional disability (7), current medical care mainly focusses on pharmacological interventions for classic symptoms which are only partially effective (8). Few non-pharmacological interventions exist to help people live well with their condition and improve quality of life (9).

Our previous work indicates that SjD patients need access to support outside of medical review appointments to empower them to self-manage symptoms of dryness, fatigue, pain and sleep disturbances (10, 11). Our qualitative work exploring potential evidence-based SjD interventions and their mode of delivery (11, 12) indicated that digital interventions, such as smartphone apps containing appropriate self-management support and behaviour change techniques (13), could be beneficial, especially for those lacking face-to-face symptom management support (12).

Self-management apps are a promising adjunct to clinical care for people with rheumatic diseases. They show high levels of usability and acceptability among young people with juvenile idiopathic arthritis (14) and favourable outcomes for rheumatoid arthritis patients (15). However, many apps designed, developed and evaluated by academics and practitioners are not made publicly available (16), and commercial apps may lack regulated, evidence-based content, such those targeting lower back pain (17). Consequently, people with rheumatic conditions, including SjD, often lack access to evidence-based apps for self-managing their symptoms outside the clinic.

The translation of rheumatology app intervention research for public benefit is further hindered by evaluation settings. Promising randomised controlled trials of self-management apps for rheumatic diseases (15) often lack external validity, not representing real-world populations and contexts. In-person trial procedures and participant payments and incentives may artificially boost engagement (18) leading to disappointing results in subsequent implementation trials. This is particularly likely for smartphone apps for which engagement is particularly low (19).

Pragmatic implementation-effectiveness trial designs can save time and improve efficiency by assessing real-world implementation alongside effectiveness (20). Early testing of adoption and engagement allows for further development and optimisation for the intended delivery setting. For self-management apps, this can mean through online marketplaces like the Apple App Store and Google Play, with increasing use by people with immune-mediated inflammatory diseases (21). These platforms have facilitated fully-remote pragmatic trials of public health apps (22, 23). To date, and to our knowledge, no self-management app for SjD has undergone feasibility testing in a pragmatic trial. A pilot feasibility design was chosen to test this complex intervention and the RCT protocol before potentially progressing to a fully powered trial (24).

In this study, we assessed the feasibility of a fully-remote and automated trial of a novel evidence-based self-management smartphone application for SjD (Sjogo). When developing the app, we followed the Medical Research Council guidance for complex intervention research (24, 25) and incorporated the European Alliance of Associations for Rheumatology recommendations on self-management in inflammatory arthritis and for developing self-management apps (26, 27). The app was based on self-determination theory (28) and informed by behaviour change techniques (29) and the previous British Society of Rheumatology iteration of the disease guideline (30). It was developed in collaboration with people with SjD who attended a series of eight design workshops (31), and informed by clinicians (rheumatologist, occupational therapist, rheumatology nurse specialist, health psychologist, sleep specialist and dentist). The app was developed with the aim of improving users' skills, knowledge and confidence in self-managing their disease (patient activation) (32) and their quality of life (QOL).

The aim of the study was to pilot and assess the feasibility of a fully-remote automated effectiveness evaluation of the Sjogo self-management app for those living with SS, as it was conducted prior to the most recent guideline (4), which uses the term SjD. Specifically, we tested trial procedures: “in app” automatic randomisation at the point of download, recruitment rates and outcome completion (attrition rates), engagement in the app through recording the number of sessions each participant engaged in and the average length of each session.

## Methods

### Study design and setting

This intervention study was conducted during 2021. Ethical approvals were obtained from Northumbria University Ethics Committee in November 2020 (reference: 120.1849). The study protocol was prospectively registered at ClinicalTrials.gov (NCT04653935). The study was an automated, double-blind, two-arm, individually randomised pilot feasibility study of the Sjogo app, to test the feasibility of the trial procedures and participant engagement. The Sjogo app (Version 1.0) was released worldwide for 8 weeks on Android Play and Apple iOS app stores in January 2021. No incentives to participate in the study were provided to ensure it was representative of a real-world setting and that people would only download it and use it if they thought it may benefit them. Potential participants who downloaded the app were guided through in-app, fully automated study procedures (eligibility screening, informed consent, symptom and QOL measures). The overarching app contained 2 sub-apps. Participants who downloaded the app were automatically randomised (simple randomisation) after completing the onboarding procedures (including eligibility screening, consent and completion of baseline measures), to an information-version (control) or the full-version of the Sjogo app. They were asked to complete outcome measures at baseline (T1), 5 (T2) and 10 weeks (T3). Push notifications were triggered, and email reminders were sent at 5 weeks and 10 weeks after downloading the app. Participants had very little contact with the researchers but could contact them via email with any technical queries. The study design and reporting were in accordance with the Consolidated Standards of Reporting Trials (CONSORT) (33) the CONSORT EHealth Checklist (34) and the CONSORT extension for pilot and feasibility trials (35).

### User involvement

People with SjD were involved at various stage of the trial, including the development of the smartphone app and its content at a series of user-centered workshops, and study design of the feasibility trial (31). We received input from people with SjD through a user-led organisation (North-East Sjögren's Syndrome Association) and a member of this organisation was a collaborator (MH).

### Recruitment

Potential participants (adults over 18 with SjD) were alerted to the trial through social media (Twitter, now known as X) and through two UK-based patient support groups (*via* email newsletter and Facebook). The term SS was used during the study, as this term was commonly used by patient groups at the time of recruitment. From the advertisements, potential participants were guided to the Google Play or Apple iOS app stores where they were able to download the Sjogo app. The app store descriptor of Sjogo explained that the app was developed by researchers for people with primary or secondary SS as part of a study. The descriptor further stated that if participants were eligible, after consenting to take part, they would be randomised to receive either a control or a full version of the app. The recruitment process started on 30th December 2020 for Android users, when the Android version of the Sjogo app was published to Google Play Store. Recruitment started for iOS users when Sjogo was published to the Apple App Store on 5th January 2021.

### Eligibility screening

Inclusion in the study was based on the following criteria: Being over 18, a diagnosis of SS by a doctor (with an option indicate either primary or secondary SS). Respondents who did not meet these criteria were thanked for their time but were unable to proceed any further within the app. Those who were under 18 and without a self-reported diagnosis of SS made by a clinician, were therefore excluded from the study.

### Consent

Participants fulfilling the inclusion criteria were guided through the in-app participant information sheet, prior to reaching the area in the app where they could provide their consent to take part in the study. Potential participants were provided with contact information (email address) of the research team and were given the opportunity to ask any questions prior to consenting. Participants were free to withdraw from the study at any time without giving a reason.

### Baseline data collection

As part of the onboarding procedures, the following baseline data was collected: sex, age, diagnosis of primary or secondary SS, years since diagnosis, device type (tablet or smartphone) and operating system (iOS or Android) and country from which the participant downloaded the app. Device identifiers were not collected.

### Intervention

#### App description

Two versions of the app were developed: a full “active” version of the app and an “information only” control (See Supplementary Material S1). In brief, the active app contained multiple behaviour change techniques (theory driven strategies or methods used to modify behaviour) (29) within the following 5 components: About Sjögren's Syndrome (symptom and lifestyle information), Energy Management, Goal Setting, Managing Difficult Times and Assertiveness and Communication Skills. It included a retrospective activity diary for logging and appraising daily activities based on energy demand. This data was compiled into weekly energy charts for users to review and plan their activities. The active app also prompted users to set SMART goals based on their values and managed through an in-app prospective planning diary. The app also included guidance for managing flare-ups, relaxation and sleep techniques, and assertive and communication exercises.

The communication style within the app text was carefully considered with validating language, key points were framed as a dialogue to facilitate interactivity and reflection, and app components were accompanied by a treatment rationale to increase user buy-in (31).

The information-only control app was created solely from the “About Sjögren's Syndrome” section of the active app, where extensive information on the condition and symptoms was provided. It contained one behaviour change technique - *Information about Antecedents* (providing information about situations, events, emotions, cognitions which reliably predict performance of behaviour) (29).

Both active and control apps were stand-alone and apart from the in-app instructions on how to navigate the app, no additional training was given to users. The app was intended to be used *ad libitum.* No recommendations were provided regarding timings, frequency, or intensity of use.

### Randomisation

Consenting users that completed baseline measures were automatically randomised to either the active or control version of the app using simple randomisation within the app (1:1). Users were aware they were being randomised but were blinded to which version of the app they were allocated to. Participants were not stratified based on age or sex. After submitting their in-app baseline measures, the following screen participants saw depended on which version they had been assigned to: users arrived at either the main page of the active version (where they could access all 5 components), or the main page of the information-only control version. Investigators were also blinded to group allocation.

### Measures

The outcomes collected were quality of life (ICECAP-A) (36), global symptom severity [EULAR Sjögren's Syndrome Patient Reported Index (ESSPRI) - including single 0–10 measurements of pain, dryness and general fatigue] (37), physical and mental fatigue (numeric visual analogue scale (VAS) based on the Profile of Fatigue and Dryness (PROFAD) (38), depression (numeric VAS), anxiety (numeric VAS), sleep (numeric VAS), impact of fatigue [Modified Fatigue Impact Scale-5-item version (MFIS-5)], Sleep Condition Indicator (SCI) (39) and patient activation (Patient Activation Measure 10 (PAM10) (32). Participants were asked to complete these measures at baseline (T1), 5-weeks (T2) and 10-weeks (T3) post app download. Further details of the selected measures are included in Supplementary Material S2. In addition, the number of participant logins to the app (sessions) and the length of each session were collated. Completion rates were calculated as the number of participants who completed all questions in all surveys at each time point.

At Weeks 5 and 10 the outcome measures became available to participants with a 2-week and 3-week data collection window at Timepoints 2 and 3 (T2 and T3) respectively to maximise engagement. The surveys were closed for analysis 13 weeks after the last user completed the onboarding and consent procedures. Completion rates were calculated as the number of participants who completed all questions in all surveys at each time point. Completion rates for T3 were calculated separately from T2 (independently not cumulatively).

### Statistical analysis

Descriptive statistics were generated for demographic variables and engagement data. Continuous variables were described using means, standard deviation and interquartile range. Categorical variables were expressed as percentages. To examine if there are significant differences in users' demographic variables between the intervention and control versions, we used chi-square test for categorical variables and t-test for continuous ones, with critical level alpha = 5%. Statistical analyses were performed using Stata v17.

## Results

### Participants

**Figure 1:**
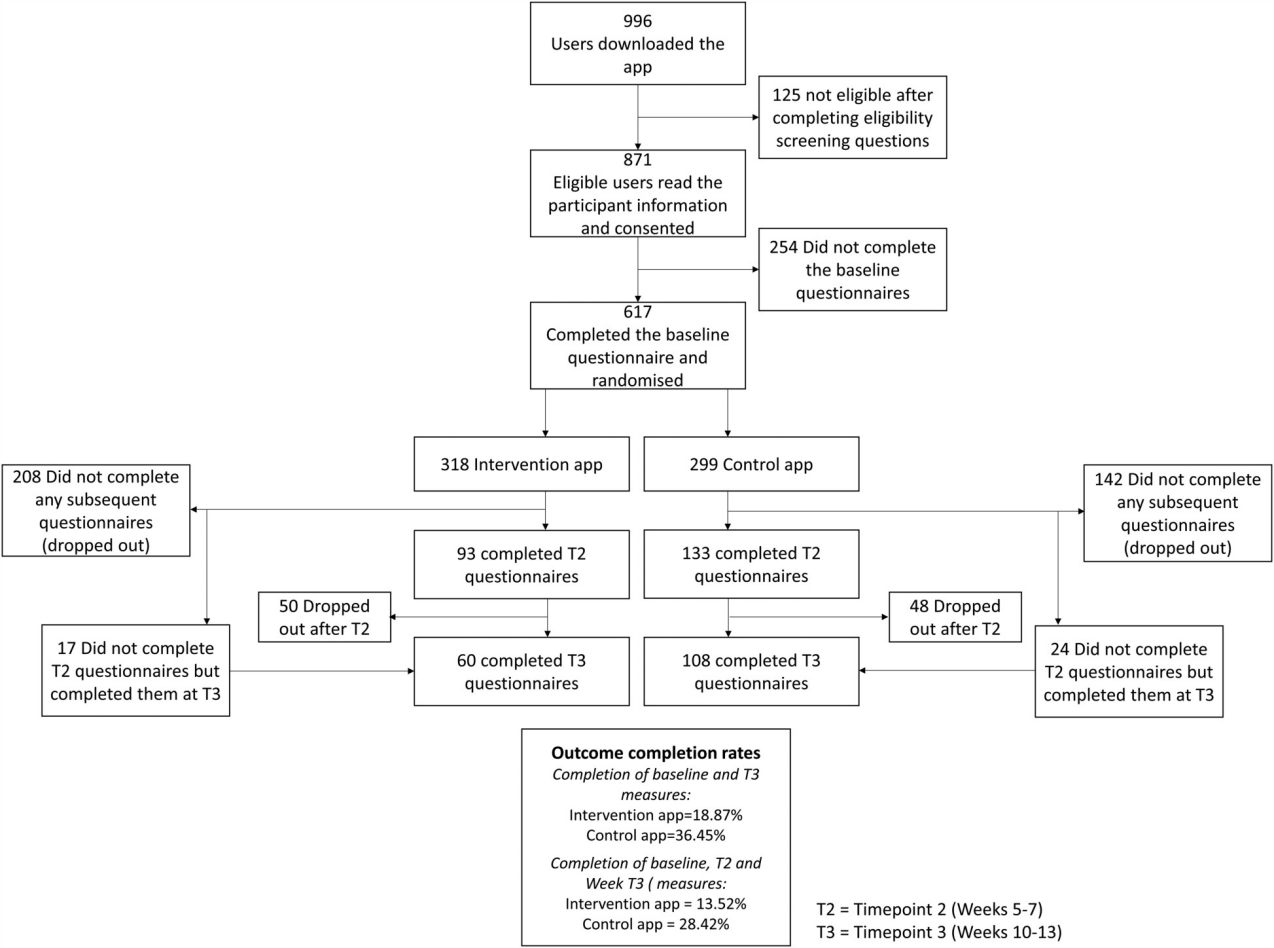
Consort diagram showing the flow of participants through the study.

Over 8 weeks in January and February 2021, 996 participants from 33 countries downloaded Sjogo to their smartphone via the Google Play and iOS Apple Stores. Of these, 871 consented to take part in the study and 617 completed the onboarding procedures, completed the baseline measures, and were randomised to the full-version of the app (*n* = 318) or control-version (*n* = 299). The flowchart of participants is shown in Figure 1.

Participants were mostly iOS users (55.11%), female (95.62%), from the UK (54.62%) or USA (28.92%) with a mean age of 50.97 (SD = 13.75; Range = 18–84). A breakdown of demographic data for participants in each condition can be seen in Table 1.

**Table 1:** In-app automatic randomisation of participants based on participants’ characteristics at baseline.

Variable	Category	Intervention		Control		*p* value^a^
		*n*	%	*n*	%	
Sex	Female	302	51.19%	288	48.81%	0.506
	Male	15	57.69%	11	42.31%	
	Other	1	100%	0	0%	
Diagnosed	Primary	280	52.34%	255	47.66%	0.312
	Secondary	38	46.34%	44	53.66%	
Operating system	Android	141	50.90%	136	49.10%	0.775
	iOS	177	52.06%	163	47.94%	
Device is tablet?	Yes	8	61.54%	5	38.46%	0.466
	No	310	51.32%	294	48.68%	
English-speaking country?*	Yes	286	51.62%	268	48.38%	0.900
	No	32	50.79%	31	49.21%	
Age**		51.19	13.45	50.74	14.08	0.683
Years since diagnosis**		6.90	7.84	6.18	6.81	0.226

* English-speaking countries: Australia (*n* = 9), Canada (*n* = 18), Great Britain (*n* = 337), Ireland (*n* = 7), New Zealand (*n* = 3), United States (*n* = 179), South Africa (*n* = 1).

** A continuous variable (years since diagnosis), we report the mean and standard deviation.

^a^ The *p*-value for the Chi-Square test for categorical variables and the *t*-test for the continuous variables (both variables are independent).

### Feasibility of in-app automatic randomisation

The automatic randomisation of participants can be seen in Table 1. Participants in each condition did not significantly differ on age, sex, diagnosis type (primary SS or secondary SS) or years since diagnosis (all *p* > 0.20). Additionally, participants in each condition did not significantly differ on operating system or device type used to access the app (*p* > 0.20).

### Outcome completion rates

Overall outcome completion rates were 36.63% at T2 and 27.39% at T3. For the full version, completion rates were 29.24% at T2 and 13.52% at T3, while the control version had rates of 44.48% at T2 and 28.42% at T3. Some participants ignored T2 prompts but went on to complete outcomes when prompted again at T3. Eighty-three participants (23.71%) were considered dropouts, having not completed any outcomes after baseline. Dropout rates were higher in the intervention group (*n* = 208) than the control group (*n* = 142). Participants who dropped out were significantly younger (*M* = 49.33, SD = 13.79) than those who remained (*M* = 53.12, SD = 13.43, *p* = 0.0003). There were no sex differences (*p* = 0.68) or differences in PAM scores (*p* = 0.46) between dropouts and those who remained. Those with higher ESSPRI scores were more likely to drop out (*M* = 6.48, SD = 1.73) compared to those who stayed (*M* = 6.10, SD = 1.73, *p* = 0.0067). No differences in individual symptom scores were observed between those who dropped out and those who remained.

### Changes in outcomes from baseline

Mean scores for each outcome measure at each time point (baseline, T2 and T3) are reported in Table 2.

**Table 2:** Mean scores for each outcome measure across three data point collections (time points 1, 2 and 3). The numbers presented are the mean ± standard deviation, and the inter-quartile range (p25 and p75 in the parenthesis).

Outcome measure	Data point collection					
	Time point 1 (baseline)		Time point 2 (week 5–7)		Time point 3 (week 10–13)	
	Intervention	Control	Intervention	Control	Intervention	Control
ICECAP-A (0–1)	0.72 ± 0.18 (0.58:0.88)	0.69 ± 0.21 (0.55:0.88)	0.72 ± 0.17 (0.60:0.88)	0.70 ± 0.20 (0.53:0.88)	0.76 ± 0.17 (0.66:0.91)	0.72 ± 0.19 (0.60:0.89)
ESSPRI (0–10)	6.33 ± 1.82 (5.00:7.67)	6.30 ± 1.65 (5.33:7.33)	6.29 ± 1.78 (5.33:7.33)	6.39 ± 1.61 (5.17:7.67)	6.24 ± 1.87 (5.50:7.50)	6.39 ± 1.60 (5.33:7.67)
PROFAD Somatic Fatigue (0–100)	58.45 ± 25.17 (42.00:78.00)	60.33 ± 23.33 (47.00:79.00)	61.43 ± 23.16 (45.00:80.00)	61.37 ± 22.64 (51.00:76.00)	61.03 ± 23.03 (55.00:75.00)	64.68 ± 19.67 (55.00:78.00)
PROFAD Mental Fatigue (0–100)	57.16 ± 26.27 (36.00:79.00)	57.28 ± 26.82 (36.00:80.00)	59.25 ± 28.32 (30.00:81.00)	55.18 ± 25.59 (37.00:75.00)	57.90 ± 27.07 (38.50:80.50)	56.51 ± 26.71 (40.00:78.00)
Depression (VAS 0–100)	40.51 ± 28.94 (15.00:64.00)	38.61 ± 30.80 (10.00:61.00)	36.39 ± 27.68 (11.50:58.50)	37.16 ± 30.33 (11.00:64.00)	36.76 ± 26.45 (12.00:55.00)	39.40 ± 31.38 (10.00:68.00)
Anxiety (VAS 0–100)	45.57 ± 28.72 (21.00:70.00)	44.65 ± 30.51 (19.00:70.00)	41.46 ± 29.22 (16.00:66.00)	43.99 ± 30.68 (15.00:70.00)	44.08 ± 27.87 (21.00:68.00)	41.80 ± 30.37 (14.00:67.00)
Difficulty Sleeping (VAS 0–100)	64.26 ± 29.68 (42.00:88.50)	60.61 ± 30.54 (35.00:86.00)	60.95 ± 29.80 (38.00:85.00)	60.29 ± 27.33 (43.00:82.00)	57.37 ± 32.12 (30.00:85.00)	55.60 ± 27.34 (37.00:76.00)
PAM-10 (0–100)	54.66 ± 11.04 (47.5:62:5)	53.83 ± 11.98 (45.00:62.50)	54.69 ± 10.36 (47.50:62.50)	53.46 ± 10.93 (45.00:62.50)	55.73 ± 10.29 (47.50:65.00)	54.91 ± 11.07 (47.50:65.00)
MFIS-5 (0–20)	12.44 ± 3.96 (10.00:15.00)	12.68 ± 3.94 (10.00:15.00)	12.03 ± 4.38 (10.00:15.00)	12.35 ± 5.36 (10.00:15.00)	12.05 ± 3.92 (10.00:14.00)	12.55 ± 3.69 (10.00:15.00)
SCI (0–32)	19.90 ± 7.13 (16:25)	20.02 ± 6.77 (16.00:25.00)	19.22 ± 6.90 (17.00:24.00)	19.25 ± 7.04 (16.00:24.00)	18.28 ± 7.47 (15.00:24.00)	19.51 ± 6.34 (17.00:23.00)

### Engagement with the Sjogo app

Participants in the intervention group logged into the Sjogo app more frequently (*M* = 11.78, SD = 37.60) than controls (*M* = 6.97, SD = 7.11, *p* = 0.03). They also used the app for a longer total duration (*M* = 57.56 min, SD = 160.5) compared to the control group (*M* = 33.35 min, SD = 32.20, *p* = 0.01).

**Figure 2:**
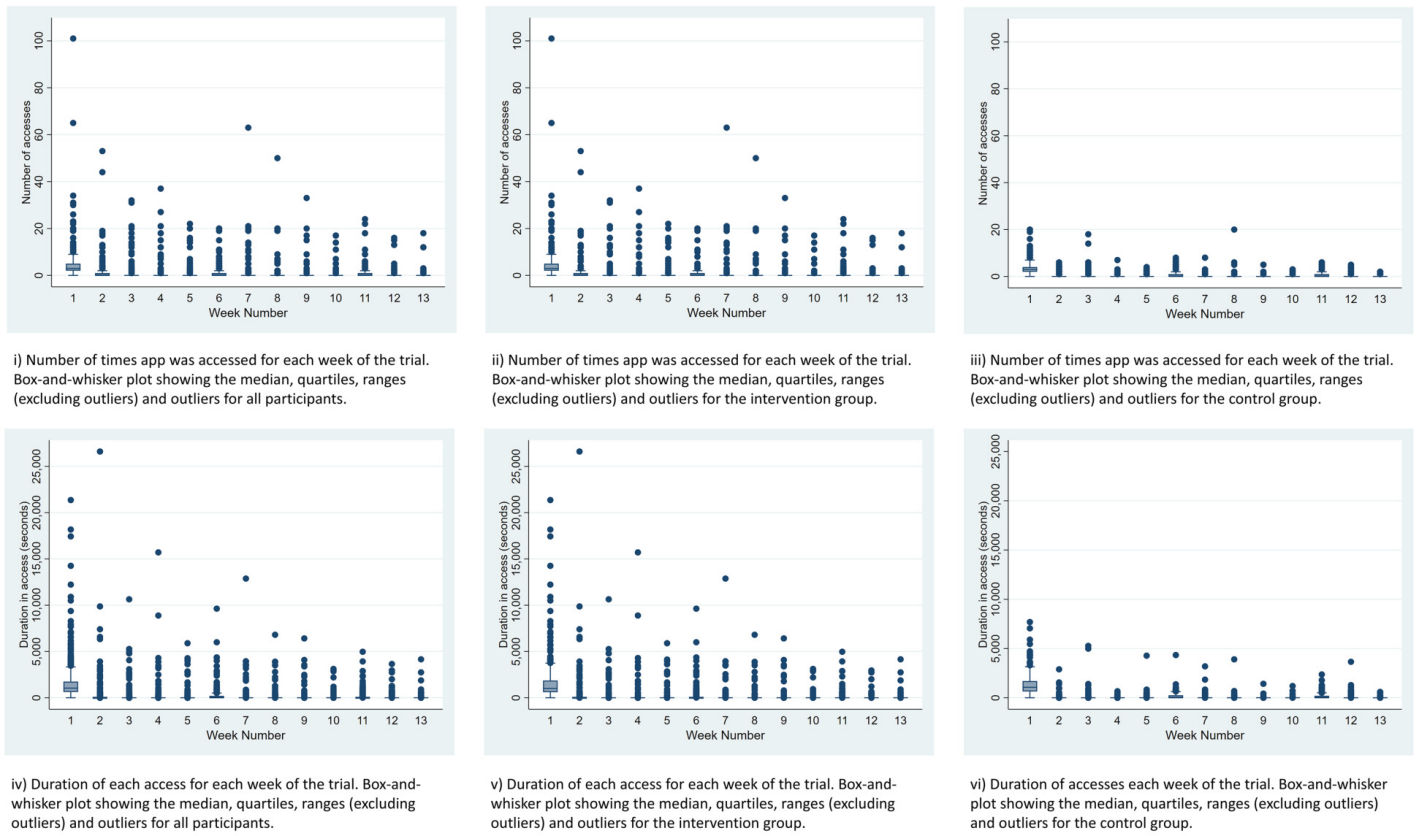
Box plots showing the number of accesses and duration of access for both apps, the intervention app and the control app for the duration of the study.

Engagement with both apps was highest in the first week (Figure 2) and declined thereafter, with the largest drop between weeks 1 and 2. In the intervention group, 318 participants accessed Sjogo an average of 4.76 times (SD = 8.06) in Week 1, including an outlier who accessed it 101 times. By Week 2, the average number of accesses fell to 1.17 times (SD = 4.56). In the control group, 299 participants accessed the information-only app an average of 3.47 times (SD = 2.75) in Week 1, dropping to 0.40 times (SD = 0.93) in Week 2.

No adverse events were reported by any participants during the study.

## Discussion

This study is the first to assess the feasibility of a fully remote effectiveness evaluation of a smartphone self-management app (Sjogo) for those living with SS.

We have demonstrated the feasibility of recruiting many participants with SS to a self-management app study via the Google Play and Apple App Stores. With minimal advertising, we achieved nearly 1,000 downloads, with over 600 users consenting and providing baseline measures. This efficient methodology demonstrates significant demand for a “direct-to-consumer” SS self-management intervention from app stores. The low entry criteria—offering a free app with information and self-management features and no in-person visits—potentially also contributed to the high recruitment (40).

The efficient recruitment suggests a future effectiveness trial could be well-powered; however, our outcome completion rates seemed low in comparison to similar studies (41). The study took place during the COVID-19 pandemic and it possible that participants were distracted with associated events which may in part have affected engagement with the study. Interestingly, completion rates at T2 and T3 were higher in the control group than in the intervention group, contrary to other self-management app studies (42). The control app contained no interactivity beyond information; however, a touchable prompt requesting participants to complete the measures, provided some interactivity (43) and may explain the greater response rates in the control group. Alternatively, simple information might have been sufficient for some participants, and it is possible they stopped engaging with the app after they had accessed it. In the intervention group, prompts may have increased user burden or led to notification fatigue (44) potentially causing participants to ignore or disable notifications.

Our fully-automated randomisation produced two well-balanced groups, similar in sex and average age of onset to the wider SS population (2). Younger participants were more likely to drop out of both study arms, possibly because the app was predominantly developed with older SS patients (31). Participants with greater ESSPRI scores were more likely to drop out, but the difference was not clinically meaningful (45), and there were no differences in individual symptom scores. Interestingly, there was no difference in PAM scores between those who dropped out and those who did not, indicating similar motivation to self-manage their condition (46), making it an unlikely reason for drop out.

Engagement was greater with the intervention app compared with the control, possibly as we co-developed it with people with SS (31) and it contained richer content, and interactive features. However, engagement sharply dropped in both groups at week two and continued to decline, similar to other studies (19). This drop could in part be due to both versions achieving desired outcomes, reducing the need for continued use (40). Alternatively, they may not have been engaging enough to overcome the typically low engagement rates for smartphone app interventions (19). Features that promote sustained engagement include gamification (47) in-app social support with peers/coaches (40) and data sharing with health professionals (48). These features were either not desired by participants during Sjogo app development (e.g., gamification) (31) or were outside the project's scope (e.g., social/health professional support). While a full trial is feasible with the current app version, incorporating social support and/or additional access to self-management coaching may potentially reduce attrition rates in a future full-scale effectiveness study. A qualitative process evaluation with a sample of participants may provide more insights which could be addressed in a future iteration of the app to try and improve engagement.

### Limitations and directions for future research

A fully-automated trial of different versions of an app provided an efficient way to implement double-blind testing. To support blinding, we took care not to reveal app features associated with only the intervention version when advertising. However, future studies could measure all users' expectations of improvement to understand the effectiveness of blinding procedures (49).

As with many pragmatic trials aiming for high ecological and external validity, there are trade-offs around control and internal validity. For example, we relied on self-report of a SS diagnosis to take part in the study, which was not verified by the researchers. Furthermore, the achieved sample was dominated by middle-aged women from English-speaking countries (mainly the UK and USA). While this generally reflects age and sex demographics of the SjD population (2), any future trial should seek to reach and recruit the broadest range of SjD participants to ensure generalisability.

In line with the CONSORT guidance for feasibility and pilot trials (35), we did not assess the app's effectiveness, as this pilot feasibility study was not powered for this. Effectiveness, in terms of impact on symptom management, in both the short and longer term, needs to be examined in further studies. Our control condition was a different version of the app that we developed, not an existing intervention like a paper booklet. The appropriate control condition for digital therapeutic apps is an ongoing debate (50). Comparing a static low-maintenance version of the app, with a complex, feature-rich version could reveal whether the latter's added effort is justified.

High loss to follow-up was observed, particularly in the intervention group, indicating possible differential attrition bias. This may have been partly due to the greater interactivity with the intervention app in comparison to the text-based control app, which may have caused an element of “notification fatigue” (51) in the intervention group. Another possible factor may have been the number of outcome measures participants were asked to complete. We have conducted qualitative interviews with participants from both groups, and the analysis of these process evaluation data will give more insights into streamlining the app and the trial procedures. However, prioritising key quality of life measures in future trials may help reduce respondent burden.

Whilst our app was developed for an English-speaking audience and mainly involved participants from high-income countries (UK and USA), future intervention development work would be required to adapt this app for diverse groups before any future context-specific feasibility study or evaluation of effectiveness.

## Conclusions

It is feasible to recruit participants to a fully remote RCT of a self-management smartphone app for SjD. While trial procedures were successful, outcome completion needs improvement. Researchers should account for high early attrition rates. Enhancing app features desired by people with SjD may boost both app engagement and outcome completion.

## Data Availability

The raw data supporting the conclusions of this article will be made available by the authors, without undue reservation.
