# Supplementary material for: A pragmatic double blind remote pilot feasibility randomised controlled trial of a self-management app for people with Sjögren disease
Source: Front Digit Health. 2025 Jun 3;7:1549093. doi: 10.3389/fdgth.2025.1549093 (PMC12170581; doi:10.3389/fdgth.2025.1549093)
Supplement: Supplementary file 1 [file Datasheet1.docx]

Supplementary Material 2

# Outcome Measures

## The ICEpop CAPability measure for Adults

The ICEpop CAPability measure for Adults (ICECAP-A) was developed as a simple measure to report capability wellbeing in adults in economic evaluations (1). It is a quality of life measure based on the capability approach framework (2) and seeks to measure what individuals can ‘do’ and ‘be’ in their lives. It is comprised of five key attributes: Stability, Attachment, Autonomy, Achievement and Enjoyment. Individuals answer one question related to each of these areas on a scale of 1-4. A capability value between 0 and 1 is then calculated for individual based on these scores, where “0” represents no capability on any attribute and “1” represents full capability on all attributes (3, 4).

## EULAR Sjogren's Syndrome Patient Reported Index

The EULAR Sjogren's Syndrome Patient Reported Index (ESSPRI) (5) was developed to assess key patient reported symptoms – pain, dryness and fatigue. A single 0-10 numerical scale measures each of these symptoms. The ESSPRI score is a global measure of the severity of symptoms experienced by the patient. It is calculated with the mean domain scores of limb pain, dryness and (somatic) fatigue.

## The Profile of Fatigue and Discomfort

The Profile of Fatigue and Discomfort (PROFAD) (6) is the first patient-reported outcome tool designed specifically for PSS patients. Initially, it was designed with 64 questions to assess different ‘facets’ of symptoms commonly experienced by PSS patients. As the original version was considered burdensome for the patient to complete, a shorter version was subsequently developed with 19 questions each reflecting a single ‘facet’ of the longer, original version. Each item is scored on an 8-point (0-7) Likert scale and an average is taken for the domain score. In this instrument, 6 questions assess somatic and mental fatigue along with a visual analogue score (VAS), ranging from 0 for absent to 100 for worst imaginable perceived fatigue levels. A score of above 2.0 and 1.8 are considered significant for the somatic fatigue and mental fatigue domains, respectively (6, 7). In this study however, only the VAS scores for somatic and mental fatigue were collected.

## Digital Numeric Visual Analogue Scales for Depression, Anxiety and Difficulties with Sleeping

Numeric VAS scales were used to measure the symptoms of depression, anxiety and sleep disturbances. Participants were asked to use sliding numeric scales to indicate how depressed and anxious they felt on average over the previous 2 weeks with 0 indicating “not at all” and 100 as “extremely anxious” or “extremely depressed”. Participants were also asked to mark on a sliding numeric VAS to represent the severity of any difficulties with sleep they had experienced over the previous two weeks, which may include getting off to sleep, staying asleep or disturbed sleep. A 0 indicated “no sleep disturbances” and 100 represented “extreme sleep disturbances”.

## Modified Fatigue Impact Scale – 5 Item

The Modified Fatigue Impact Scale – 5 item (MFIS-5) (8) is a modified version of the 21-item Modified Fatigue Impact Scale, which in turn was modified from the 40-item Fatigue Impact Scale (FIS), which measures the effect of fatigue on quality of life in people with chronic diseases (9, 10). The MFIS-5 measures the impact of fatigue on cognitive, physical and psychosocial function. It contains A 5-point Likert scale is used to rate each item from 0 (which indicates “never”) to 4 = (which indicates “almost always”), yielding a total score ranging from 0 to 20, with higher scores suggest a greater fatigue impact (8).

## Sleep Condition Indicator

The Sleep Condition Indicator (SCI) (11) is an 8-item measure which evaluates insomnia. The measure includes questions on the following: time taken to get to sleep, remaining asleep, sleep quality, personal functioning during the day, daytime performance, nights per week affected by the sleep problem, the extent troubled by poor sleep, and the duration of the sleep problem. Each item is scored on a 5-point scale (0–4), with a possible total score range from 0 to 32. Higher values are indicative of better sleep. Scores can also be transformed into a 0–10 SCI range by dividing the total by 3.2. The measure has good psychometric properties, it can be used to screen for insomnia, and determine whether a reliable change was achieved following intervention (11-13).

## Patient Activation Measure-10

The 10-item Patient Activation Measure (PAM-10) is a 10-item measure of patient knowledge, skill, and confidence in managing one’s own health or healthcare (14, 15). Higher scores are associated with improved health outcomes, decreased health costs and greater satisfaction in healthcare (16). PAM-10 scores range from 0–100 with a lower score indicating lesser knowledge, skill and confidence in managing one’s own health and a higher score indicating greater confidence and ability for self-management.

## References

1. Al-Janabi H, N Flynn T, Coast J. Development of a self-report measure of capability wellbeing for adults: the ICECAP-A. Quality of Life Research. 2012;21(1):167-76.

2. Sen A. Capability and Well-Being In M Nussbaum and A Sen (eds) The Quality of Life. New York. 1993.

3. Rencz F, Mitev AZ, Jenei B, Brodszky V. Measurement properties of the ICECAP-A capability well-being instrument among dermatological patients. Quality of life research : an international journal of quality of life aspects of treatment, care and rehabilitation. 2022;31(3):903-15.

4. Flynn TN, Huynh E, Peters TJ, Al-Janabi H, Clemens S, Moody A, et al. Scoring the Icecap-a capability instrument. Estimation of a UK general population tariff. Health economics. 2015;24(3):258-69.

5. Seror R, Ravaud P, Mariette X, Bootsma H, Theander E, Hansen A, et al. EULAR Sjogren's Syndrome Patient Reported Index (ESSPRI): development of a consensus patient index for primary Sjogren's syndrome. Annals of the rheumatic diseases. 2011;70(6):968-72.

6. Bowman SJ, Booth DA, Platts RG, Group UKSsI. Measurement of fatigue and discomfort in primary Sjogren's syndrome using a new questionnaire tool. Rheumatology (Oxford). 2004;43(6):758-64.

7. Segal B, Thomas W, Rogers T, Leon JM, Hughes P, Patel D, et al. Prevalence, severity, and predictors of fatigue in subjects with primary Sjogren's syndrome. Arthritis Rheum. 2008;59(12):1780-7.

8. D’Souza E. Modified Fatigue Impact Scale – 5-item version (MFIS-5). Occupational Medicine. 2016;66(3):256-7.

9. Larson RD. Psychometric properties of the modified fatigue impact scale. International journal of MS care. 2013;15(1):15-20.

10. Fisk JD, Pontefract A, Ritvo PG, Archibald CJ, Murray TJ. The impact of fatigue on patients with multiple sclerosis. The Canadian journal of neurological sciences Le journal canadien des sciences neurologiques. 1994;21(1):9-14.

11. Espie CA, Kyle SD, Hames P, Gardani M, Fleming L, Cape J. The Sleep Condition Indicator: a clinical screening tool to evaluate insomnia disorder. BMJ Open. 2014;4(3):e004183.

12. Espie CA, Farias Machado P, Carl JR, Kyle SD, Cape J, Siriwardena AN, et al. The Sleep Condition Indicator: reference values derived from a sample of 200 000 adults. Journal of sleep research. 2018;27(3):e12643.

13. Wong ML, Lau KNT, Espie CA, Luik AI, Kyle SD, Lau EYY. Psychometric properties of the Sleep Condition Indicator and Insomnia Severity Index in the evaluation of insomnia disorder. Sleep medicine. 2017;33:76-81.

14. Hibbard JH, Stockard J, Mahoney ER, Tusler M. Development of the Patient Activation Measure (PAM): conceptualizing and measuring activation in patients and consumers. Health services research. 2004;39(4 Pt 1):1005-26.

15. Hibbard JH, Mahoney ER, Stockard J, Tusler M. Development and testing of a short form of the patient activation measure. Health services research. 2005;40(6 Pt 1):1918-30.

16. Hibbard JH, Greene J. What the evidence shows about patient activation: better health outcomes and care experiences; fewer data on costs. Health affairs (Project Hope). 2013;32(2):207-14.
