# Supplementary material for: A pragmatic double blind remote pilot feasibility randomised controlled trial of a self-management app for people with Sjögren disease
Source: Front Digit Health. 2025 Jun 3;7:1549093. doi: 10.3389/fdgth.2025.1549093 (PMC12170581; doi:10.3389/fdgth.2025.1549093)
Supplement: Supplementary file 2 [file Table1.docx]

Supplementary Materials 1

## Supplementary Table 1 showing description of the intervention (Sjogo) and control app (Information Only) components

| Component number | Component name | Content/app feature | Behaviour Change Technique Number (1) | Sjogo | Information only |
| --- | --- | --- | --- | --- | --- |
|  | Why manage activity | Full screen text content to provide the scientific justification behind activity management and to validate the need to pace activities. | 4.2 Information about antecedents | **✓** | **X** |
|  | Activity Diaries:    Planned Diary    Reflective Diary | Full screen fixed-grid layout with input chips and a floating action button to input blocks of time. Opens a full screen dialogue for users to colour code activities according to how much effort they take. | 1.4 Action planning  2.3 Self-monitoring of behaviour | **✓** | **X** |
|  | Activity History | Colour-coded pie charts to demonstrate past day activity break down, rests, and how they influence crashes | 2.3 Self-monitoring of behaviour  2.4 Self-monitoring of outcome(s) of behaviour | **✓** | **X** |
|  | Managing Activity | Full screen dialogue with introductory text. Floating action buttons which link to additional content. | 4.1 Instruction on how to perform a behaviour | **✓** | **X** |
|  | Boom and Bust | Full screen text content and image containers with page control navigation. Floating action button to open floating sheet displaying ‘My Data’ for reference. | 4.2 Information about antecedents | **✓** | **X** |
|  | Baseline | Full screen text content and image containers with page control navigation. Floating action button to open floating sheet displaying ‘My Data’ for reference. | 4.2 Information about antecedents | **✓** | **X** |
|  | Prioritising | Full screen text content with page control navigation. Floating action button to open floating sheet displaying ‘My Data’ for reference. | 4.1 Instruction on how to perform a behaviour | **✓** | **X** |
|  | Planning rests | Full screen text content and image containers with page control navigation. Floating action button to open floating sheet displaying ‘My Data’ for reference. | 4.1 Instruction on how to perform a behaviour | **✓** | **X** |
|  | Pacing | Full screen text content and image containers with page control navigation. Floating action button to open floating sheet displaying ‘My Data’ for reference. | 4.1 Instruction on how to perform a behaviour  12.2 Restructuring the social environment | **✓** | **X** |
|  | Switching tasks | Full screen text content and image containers with page control navigation. Floating action button to open floating sheet displaying ‘My Data’ for reference. | 4.2 Information about antecedents | **✓** | **X** |
|  | Energisers and drainers | Full screen text content with page control navigation. Floating action button to open floating sheet displaying ‘My Data’ for reference. | 4.1 Instruction on how to perform a behaviour | **✓** | **X** |
|  | Activity grading | Full screen text content with page control navigation. Floating action button to open floating sheet displaying ‘My Data’ for reference. | 4.1 Instruction on how to perform a behaviour | **✓** | **X** |
|  | Goals | Full screen tab to input personal goals. Contains a floating action button which opens a full-screen dialogues for users to input life area goals. | - 1. Goal setting | **✓** | **X** |
|  | What to do during a crash | Full screen multi-page text content | 4.2 Information about antecedents | **✓** | **X** |
|  | Relaxation techniques | Full screen multi-page text content | 4.1 Instruction on how to perform a behaviour | **✓** | **X** |
|  | Dealing with setbacks | Full screen multi-page text content | 4.2 Information about antecedents | **✓** | **X** |
|  | Warning | Full screen multi-page text content: counter indications for engaging with the sleep content | 4.2 Information about antecedents | **✓** | **X** |
|  | What is stimulus control and how might it help | Full screen multi-page text content | 4.2 Information about antecedents | **✓** | **X** |
|  | How do I do stimulus control | Full screen text content with expanded content available on user interaction. | 4.1 Instruction on how to perform a behaviour | **✓** | **X** |
|  | Common questions | Full screen text content with expanded content available on user interaction. | 4.2 Information about antecedents | **✓** | **X** |
|  | Stimulus control-alternatives and adaptations | Full screen multi-page text content | 4.1 Instruction on how to perform a behaviour | **✓** | **X** |
|  | What are cognitive control techniques | Full screen text content | 4.2 Information about antecedents, 5.6 Information about emotional consequences | **✓** | **X** |
|  | Cut-off time | Full screen text content | 8.4 Habit reversal  8.2 Behaviour substitution | **✓** | **X** |
|  | Cognitive control diary | Full screen multi-page text content | 5.4 Monitoring of emotional consequences | **✓** | **X** |
|  | Cognitive distraction | Full screen multi-page text content | 12.4 Distraction | **✓** | **X** |
|  | Why assertiveness | Full screen multi-page text content | 5.6 Information about emotional consequences | **✓** | **X** |
|  | Communication cards | Floating action buttons which link to full screen text content relating to related explanatory text | 4.2 Information about antecedents  9.1. Credible source | **✓** | **X** |
|  | 10 rules of assertiveness | Full screen text content | 4.2 Information about antecedents | **✓** | **X** |
|  | What’s the alternative? | Layered full screen multi-page text content | 5.3. Information about social and environmental consequences | **✓** | **X** |
|  | Assertiveness tools | Introductory text with floating action buttons which link to full screen text content | 4.1 Instruction on how to perform a behaviour | **✓** | **X** |
|  | Sjogren’s Syndrome | Accessed via information drawer (hamburger menu), with floating action buttons which link to additional text content | 4.2 Information about antecedents | **✓** | **✓** |
|  | Fatigue | Accessed via information drawer (hamburger menu), with floating action buttons which link to additional text content | 4.2 Information about antecedents | **✓** | **✓** |
|  | Dryness | Accessed via information drawer (hamburger menu), with floating action buttons which link to additional text content | 4.2 Information about antecedents | **✓** | **✓** |
|  | Sleep | Accessed via information drawer (hamburger menu), with floating action buttons which link to additional text content | 4.2 Information about antecedents | **✓** | **✓** |
|  | Pain | Accessed via information drawer (hamburger menu), with floating action buttons which link to additional text content | 4.2 Information about antecedents | **✓** | **✓** |
|  | Managing Dryness | Accessed via information drawer (hamburger menu), and related floating action button. Provides additional full screen multi-page text content | 4.2 Information about antecedents | **✓** | **✓** |
|  | Managing Fatigue | Accessed via information drawer (hamburger menu), and related floating action button. Provides additional full screen multi-page text content | 4.2 Information about antecedents | **✓** | **✓** |
|  | Managing Pain and discomfort | Accessed via information drawer (hamburger menu), and related floating action button. Provides additional full screen multi-page text content | 4.2 Information about antecedents | **✓** | **✓** |
|  | Managing Sleep | Accessed via information drawer (hamburger menu), and related floating action button. Provides additional full screen multi-page text content | 4.2 Information about antecedents | **✓** | **✓** |
|  | Eye dryness | Accessed via information drawer (hamburger menu), and related floating action button. Provides additional full screen multi-page text content | 4.2 Information about antecedents | **✓** | **✓** |
|  | Oral hygiene | Accessed via information drawer (hamburger menu), and related floating action button. Provides additional full screen multi-page text content | 4.2 Information about antecedents | **✓** | **✓** |
|  | Vaginal dryness | Accessed via information drawer (hamburger menu), and related floating action button. Provides additional full screen multi-page text content | 4.2 Information about antecedents | **✓** | **✓** |
|  | Sleep tips | Accessed via information drawer (hamburger menu), and related floating action button. Provides additional full screen multi-page text content | 4.2 Information about antecedents | **✓** | **✓** |
|  | Notes | Accessed via information drawer (hamburger menu). Floating action button to generate user specified fields which contain unstructured notes | N/A | **✓** | **✓** |
|  | About this app | Accessed via information drawer (hamburger menu). Full screen text featuring how the app was created, including a link to a website with authors’ pictures, institutional affiliations and bio | 9.1. Credible source | **✓** | **✓** |
|  | Study participation | Accessed via information drawer (hamburger menu). Full screen text featuring information about the study, participation and a link to withdraw consent. | N/A | **✓** | **✓** |
|  | Tutorial | Accessed via information drawer (hamburger menu). Link to tutorial video. | N/A | **✓** | **✓** |
|  | Covid 19 | Accessed via information drawer (hamburger menu). Full screen multi-page text featuring information about COVID-19. | N/A | **✓** | **✓** |
|  | Privacy policy | Accessed via information drawer (hamburger menu). Full screen text featuring information about the study’s privacy policy. | N/A | **✓** | **✓** |
|  | Contact us | Accessed via information drawer (hamburger menu). Full screen multi-page text featuring information about how to contact the research team. | N/A | **✓** | **✓** |

1. Michie S, Richardson M, Johnston M, Abraham C, Francis J, Hardeman W, et al. The behavior change technique taxonomy (v1) of 93 hierarchically clustered techniques: building an international consensus for the reporting of behavior change interventions. Ann Behav Med. 2013;46(1):81-95.

**
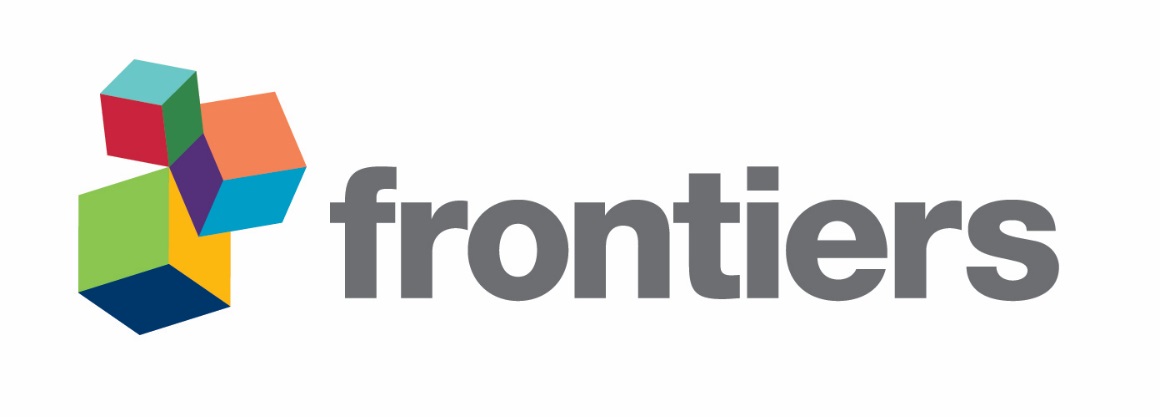
**
